# Concurrent medical conditions among pregnant women - ignore at their peril: report from an antenatal anesthesia clinic

**DOI:** 10.1186/s13584-018-0210-7

**Published:** 2018-03-19

**Authors:** Carolyn F. Weiniger, Sharon Einav, Uriel Elchalal, Vladislav Ozerski, Daniel Shatalin, Alexander Ioscovich, Yehuda Ginosar

**Affiliations:** 1 Hadassah Hebrew University Medical Center, Jerusalem, Israel; 2 Division of Anesthesia, Pain and Critical Care, Tel-Aviv Sourasky Medical Center, Tel-Aviv, Israel; 3 Shaare Zedek Medical Center and Hebrew University Faculty of Medicine, Jerusalem, Israel; 4 Washington University Medical Center, St Louis, MO USA

**Keywords:** Antenatal, Anesthesia, Comorbid, Maternal, Medical

## Abstract

**Background:**

Care of pregnant women with concurrent medical conditions can be optimized by multidisciplinary antenatal management. In the current study we describe women with concurrent medical conditions who attended our antenatal anesthesia clinic over a 14-year period, 2002–2015 and, based on the findings, we suggest new policies, strategies and practices to improve antenatal care.

**Methods:**

In 2002, an antenatal anesthesia clinic was established in Hadassah Medical Center. Each consultation focused on the concurrent medical condition. A written anesthesia strategy according to the medical condition and its anesthesia considerations was discussed and given to the patient. Data regarding clinic visits were recorded.

**Results:**

A total of 451 clinic women attended the antenatal anesthesia clinic. Maternal age was 31.7 ± 6.0 years (mean ± SD), with gestational age of pregnancy 33.0 ± 5.4 weeks at the clinic visit. Musculoskeletal conditions (23% of all the women seen) were the most frequent concurrent conditions, followed by anesthesia related concerns 20%, neurologic conditions 19%, and cardiac conditions 15%. Women were provided plans that were deliberated carefully rather than being concocted during labor.

**Conclusions:**

A wide range of concurrent medical conditions was seen in the antenatal anesthesia clinic, however fewer women attended the clinic than expected according to known population frequencies of concurrent medical conditions. Women with concurrent medical conditions should have labor and anesthesia plans considered during the nine months of pregnancy, prior to delivery, and hospitals should have a means of obtaining this information in a timely manner. Finally, there is a need to develop additional antenatal anesthesia clinics.

## Background

Traditional causes of maternal death such as hypertension and hemorrhage have been overtaken in the United Kingdom (UK) and the United States (US), and some developing countries by deaths due to concurrent medical conditions such as cardiac disease [1–3]. Furthermore, the UK Saving Lives project reported that multiple antenatal opportunities to improve care were missed, particularly among women with concurrent medical conditions, and that “fragmented”, uncoordinated care contributed to maternal mortality [2]. In a recent Brazilian study, significant cardiac disease that contributed to morbidity during labor was known in 83% of women prior to pregnancy [4].

At the same time, the trend to higher maternal mortality due to concurrent medical conditions is paralleled by increased prevalence of maternal morbidities [3, 5, 6]. Thus recent evidence suggests that national maternal mortality rates are insufficient to determine improvements required for maternal care [2, 6]. Maternal morbidity reports are now considered by the WHO to be a more valuable indicator of maternal care [6].

Acknowledging the greater obstetric and medical comorbidity among pregnant women with concurrent medical conditions, [3, 7] the American Society of Anesthesiologists’ “Practice Guidelines for Obstetric Anesthesia” endorse multidisciplinary care these women [8]. Since care of pregnant women with concurrent medical conditions can be optimized by multidisciplinary antenatal management [9], in 2017 the American College of Obstetrics and Gynecologists (ACOG) generated a Practice Bulletin containing a list of concurrent conditions that require anesthesia consultation during pregnancy [10].

In order to facilitate optimal anesthesia care for pregnant women with concurrent medical conditions, antenatal anesthesia clinics have been established in several countries, including Israel [11–17]. There are currently seven antenatal anesthesia clinics operating in Israel. These clinics play an increasingly important role in planning anesthesia care for delivery and the peripartum period.

In this study, we describe the frequency of the concurrent medical conditions reported among pregnant women attending the antenatal anesthesia clinic of a large tertiary medical center in Jerusalem over a 14-year period (2002–2015); and we report the anesthesia plans made for labor and delivery. From this experience, we derive some lessons that should be useful for improving care of women during pregnancy and delivery and to improve outcomes.

## Methods

### Design and ethical considerations

This study was comprised of prospectively collected clinic assessments in addition to retrospectively retrieved delivery details. The study was performed with Institutional Review Board approval (waiver from written informed consent, 0121–15-HMO Hadassah Medical Center).

### Clinical setting

All Israeli citizens are mandatory members of one of four Health Management Organizations (HMOs). Nationwide medical care is subsidized for all citizens via taxation and HMO insurance costs. Pregnant women are followed throughout pregnancy either in their HMO or in dedicated government funded Mother and Baby clinics (Tipat Halav). In 2011 the Israeli Ministry of Health (MOH) issued a statement listing 23 medical conditions that require special attention during the antenatal period [18]. Women with any of these conditions, or a fetal condition, are directed to receive antenatal care in their HMO, not in the Mother and Baby clinics [18]. Referrals to specialist care are made at the discretion of the HMO Obstetrician. These may include referral to an expert clinic, to a hospital for further investigations or even antenatal hospitalization if necessary.

The Hadassah Medical Center high risk antenatal anesthesia clinic was established in 2002. This clinic runs once weekly with referrals sought actively by mailing both the local gynecologists serving different HMO plans and family doctors. These approaches have been repeated periodically since the clinic’s inception. In addition, a flyer was produced and sent to all HMOs serving the Jerusalem district, accompanied by a request to place them in a location highly visible to all pregnant women visiting the HMO. Pregnant women made an appointment for the antenatal anesthesia clinic using the hospital appointment system, and received a referral from their HMO that paid for the clinic consultation. The clinic was staffed by a specialist Obstetric Anesthesiologist or Fellow supervised by a specialist Obstetric Anesthesiologist and clinic hours were designated as protected clinical time.

### Study population

We included pregnant women with concurrent medical conditions and any pregnant woman seeking an anesthesia consultation prior to the labor and delivery, who attended the Hadassah Medical Center antenatal anesthesia clinic.

### Clinic consultation components

Each consultation discussed the woman’s general history including demographics, gestational age at consult, referring physician/midwife and HMO affiliation. The obstetric history taken included gravidity, parity, current and past obstetric history such as mode of delivery and complications in prior pregnancies and delivery plans for current pregnancy. The medical history taken included details of the cause of referral and current medications. Each consultation yielded a written plan for neuraxial block recommendations for labor, cesarean delivery anesthesia recommendations (elective or urgent), and medication strategies (e.g. stopping anticoagulation in order to enable neuraxial anesthesia) [19]. This plan included appraisal of risk if general anesthesia would be required for cesarean delivery, additional consultations and investigations if required, and the likely need for intensive care; postpartum recommendations included postpartum monitoring and venous-thromboembolism prophylaxis. Detailed notes were scanned (2002–2010) or written (2010–2015) into the patient’s electronic medical record (EMR) in order to ensure their availability whenever the women presented for medical care. A print-out of the clinic visit findings and recommendations was given the pregnant woman.

### Data retrieval

Clinic attendees were identified by a unique code identifying a visit to the antenatal anesthesia consultation service. From the period 2002–2010, data regarding clinic visits were entered into a spreadsheet, Excel 95, Windows 98, Microsoft. Data from the period 2010–2015 were retrieved from the EMR. All data were retrieved by two of the investigators (DS and VO), and were entered into a computer database (Excel 95, Windows 98, Microsoft).

The data that were entered into the spreadsheet (2002–2009) and/or the EMR (2010–2015) included maternal demographic and obstetric characteristics noted in the clinic consultation data; medical history; obstetric conditions including current pregnancy details e.g. twins, presentation, planned delivery mode; and an anesthesia plan. Actual delivery details were retrieved for the later cohort, 2010–2015 from the labor and delivery EMR.

The primary study outcome was the frequency of conditions according to previously described classifications [15–17].

Secondary study outcomes included:

Anesthesia performed for labor and delivery for the EMR cohort, 2010–2015.

Adherence to recommendations for anesthesia plan for the EMR cohort, 2010–2015.

Mode of delivery, according to medical condition for the EMR cohort, 2010–2015.

### Statistical analysis

We present descriptive statistics including variable frequencies, presented as numbers (percentages) and mean (standard deviation) and range, calculated using IBM SPSS version 21.0.0 for Windows (IBM Corp. Armonk, NY).

## Results

**Fig. 1:**
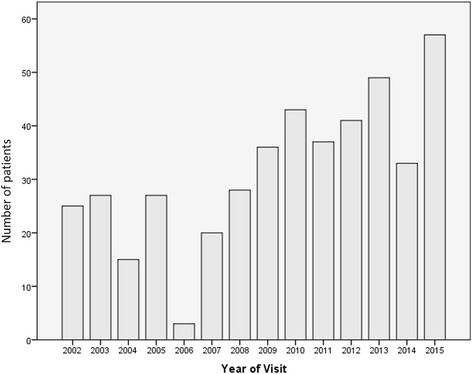
Number of clinic visits recorded according to year

During the period 2002–2015 there were a total of 451 clinic visits at Hadassah Medical Center. The clinic visits according to year are presented in Fig. 1.

The baseline characteristics are presented in Table 1. The women seen at the antenatal anesthesia clinic were slightly older mean ± SD age 31.7 ± 6.0 years than the overall population of pregnant women, 30.1 ± 5.7 who delivered in Hadassah during the study period. The gestational age of pregnancy at the clinic visit was 33.0 ± 5.4 weeks. The women seen at the antenatal anesthesia clinic had a mean ± SD [range] gravidity 2.4 ± 1.8 [1–3], and parity 1.2 ± 1.6 [0–2]; slightly lower than the overall population who had gravidity 3.3 ± 2.4 [0–24] and parity 1.8 ± 2.0 [0–17].

**Table 1:** Maternal characteristics of the 2002–2015 study cohort

	All Patients *N* = 451
Maternal age at clinic visit, year (mean ± SD)	31.7.6 ± 6.0
Gravidity at clinic visit (mean ± SD [range])	2.4 ± 1.8 [1–3]
Parity at clinic visit, (mean ± SD [range])	1.2 ± 1.6 [0–2]
Gestational age at clinic visit, weeks (mean ± SD)	33.0 ± 5.4
Referring health fund	
Maccabi	119 (26.3%)
Meuhedet	65 (26.3%)
Leumit	17 (3.8%)
Clalit	48 (10.6%)
Unknown/Self-Pay	69 (15.2%)
Data are missing for 131 women	
Anesthesia modality recommended	
No limitations	283 (62.5%)
Regional recommended	43 (9.5%)
Regional contraindicated	31 (6.9%)
Depends on lab/status	68 (15.1%)
General anesthesia	24 (5.3%)
Unknown	2 (0.4%)

**Fig. 2:**
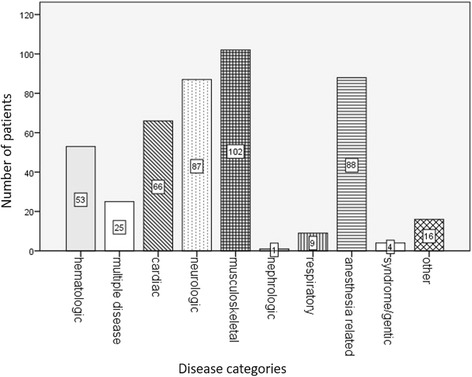
The bar chart shows the attendances to the clinic according to medical disease classification for all women, 2002–15

The conditions leading the women to attend the clinic are presented in Fig. 2. Most frequently seen were musculoskeletal conditions (23%, of all women seen). As examples, these included symptomatic disc herniation (*n* = 49), major scoliosis corrective surgery or uncorrected scoliosis (*n* = 20), cervical-spine injury (*n* = 7), other major back surgery after motor vehicle accident (*n* = 3), and spina bifida occulta (*n* = 3) among others. This was followed by anesthesia related concerns (20% of the total), including previous anaphylaxis (*n* = 17), prior failed or difficult block (*n* = 30), previous accidental dural puncture (*n* = 5), previous difficult intubation (n = 3), tattoo (n = 3). Neurologic conditions (19%) included cerebral tumor (past or current) (*n* = 10), neuropathy (*n* = 9), prior cerebrovascular accident (n = 7), pseudo-tumor cerebri (*n* = 6), multiple sclerosis (n = 6), myasthenia gravis (*n* = 5), epilepsy (n = 5), arterio-vascular malformation (n = 3), cavernoma (n = 3) and Arnold-Chiari malformation (n = 1). Cardiac conditions (15%) included congenital heart disease (CHD) (*n* = 21), arrhythmia (*n* = 11) and significant valve disease (not rheumatic fever (RF) or CHD) (n = 11), significant proximal aortic disease (*n* = 4), cardiomyopathy (n = 2), RF (n = 2), Hematologic conditions accounted for 53 (12%) of the clinic visits for conditions and included: thrombocytopenia (gestational or other) (*n* = 13), factor deficiency (n = 11), von Willebrand disease (*n* = 5), idiopathic thrombocytopenia (n = 4), systemic lupus erythema (n = 4), other reason for anticoagulation therapy (n = 5). Twenty-nine women had multiple organ disease/syndromes for example: Carnitine palmitoyl deficiency type II, multiple vascular stenosis (carotid artery, renal artery, mesenteric artery), portal hypertension (esophageal varices, splenomegaly), Marfan syndrome, ankylosing spondylitis (with multiple arthritic expressions including limited mouth opening).

Table 2 shows the recommended versus actual anesthesia/analgesia provided for labor and delivery for 260 women attending the clinic 2010–2015. Eleven women were advised to avoid neuraxial block due to hematologic (*n* = 4), musculoskeletal (*n* = 3) and neurological (n = 3) conditions. One woman with such a neurological condition received two prior epidurals for labor, and after the second, she had a sensory deficit that did not recover. Thirty-five women were advised that the possibility of neuraxial block for labor was dependent on their status at that time. For example, 16 women with hematologic conditions were told the decision for neuraxial block was dependent on her thrombocytes at the time of block request. All these women were given written alternative analgesia plans that included intravenous opioids via patient-controlled analgesia, and nitrous oxide.

**Table 2:** Recommended versus actual anesthesia/analgesia provided for labor and delivery, 2010–2015

Anesthesia performed for labor and delivery	No limitations (*N* = 108)	Neuraxial block recommended (*N* = 29)	Neuraxial block contraindicated (*N* = 8)	General anesthesia (*N* = 15)	Depends on lab/status (*N* = 35)
Neuraxial block performed	87 (81.3%)	27 (93.1%)	1 (12.5%)	0	22 (47.8%)
Nitrous oxide/Intravenous analgesia performed	1 (0.9%)	0	0	0	2 (4.3%)
General anesthesia performed	4 (3.7%)	1 (3.4%)	4 (50.0%)	13 (86.7%)	5 (10.9%)
No anesthesia/Analgesia performed	16 (15.0%)	1 (0.0%)	3 (37.5%)	2 (13.3%)	6 (13.0%)

For 63 women the labor analgesia/anesthesia received was not known

Among the 31 women (2010-2015) with a hematologic condition, five (16.7%) had no limitation for the neuraxial block, and four (12.9%) were advised to avoid neuraxial block. Other details regarding anesthesia plans are presented in Table 3.

**Table 3:** Anesthesia performed according to disease classification (2010-2015)

	Neuraxial block	Intravenous/Inhalational	General anesthesia	Not performed	Missing data
Hematologic (*N* = 31)	10 (32.3%)	1 (3.2%)	7 (22.6%)	7 (22.6%)	6 (19.4%)
Cardiovascular (*N* = 32)	21 (65.6%)	0	1 (3.1%)	1 (3.1%)	9 (28.1%)
Neurologic (*N* = 36)	17 (47.2%)	0	5 (13.9%)	4 (11.1%)	10 (27.8%)
Musculoskeletal (*N* = 71)	32 (45.1%)	1 (1.4%)	7 (9.9%)	11 (15.5%)	20 (28.2%)
Respiratory (*N* = 2)	2 (100%)	0	0	0	0
Nephrologic (*N* = 1)	0	0	0	0	1 (100%)
Anesthesia (*N* = 45)	26 (57.8%)	1 (2.2%)	1 (2.2%)	4 (8.9%)	13 (28.9%)
Syndrome/Genetic (*N* = 4)	3 (75.0%)	0	0	0	1 (25.0%)
Multiple disease (*N* = 38)	27 (71.1%)	0	6 (15.8%)	1 (4.5%)	4 (18.2%)

We present some case vignettes to illustrate the cases seen in the antenatal anesthesia clinic.

Case 1, Musculoskeletal condition: A 22 year old woman was seen at 37 weeks’ gestation. She was scheduled for birth in the Hadassah natural birthing center, and was referred to the antenatal anesthesia clinic by the midwife due to severe scoliosis. The patient revealed a previously unrecorded very strong family history of scoliosis and malignant hyperthermia in one of her siblings. Due to signs consistent with central core disease (high arched palate plus scoliosis) and a highly elevated creatinine phosphokinase she was considered susceptible for malignant hyperthermia. Her delivery plan included epidural analgesia placed in early labor using ultrasound guidance, and preparation for gas free general anesthesia if needed for emergency cesarean delivery. She received the epidural for labor as planned. Without the antenatal assessment, the susceptibility for malignant hyperthermia would not have been identified. Subsequent muscle biopsy confirmed the malignant hyperthermia diagnosis.

Case 2, Cardiac condition: A 35 year old woman was seen at 37 weeks’ gestation. She had undergone repair of Tetralogy of Fallot at age four, and follow up surgery at age 15. Currently, she had mild tricuspid regurgitation without right ventricular dilatation, and mild-moderate aortic regurgitation. She also had thalassemia minor with hemoglobin below 9.9 g/dl. Clinically she was well: New York Heart Association Classification 2. The joint consultation plan (anesthesiologist/cardiologist/obstetrician) was for normal vaginal delivery with epidural analgesia if she required it; and she did have uneventful vaginal delivery with epidural analgesia.

Case 3, Hematologic condition: A 22 year old woman was seen at week 38. She had von Willebrand disease (27% factor) which was discovered after persistent epistaxis and bruising. She was advised to consider Desmopressin, a drug with several actions, including the ability to promote the release of von Willebrand factor, before labor. Her written anesthesia plan for labor listed appropriate alternatives to epidural analgesia, after a full discussion with her. This plan included: Transcutaneous nerve stimulation, nitrous oxide 50:50 or intravenous (IV) fentanyl patient-controlled analgesia (PCA). The written plan requested notification of the anesthesiologist when she presented to the labor ward to allow the plans to be updated and contextualized to the actual labor. She delivered vaginally without analgesia.

## Discussion

The antenatal anesthesia clinic for pregnant women with concurrent medical conditions in our center has changed practice. Prior to establishment of the clinic, delays in care occurred while additional information was sought to provide optimal anesthesia given the concurrent medical conditions. Now, pregnant women with concurrent medical conditions who have attended the clinic, present to the labor ward with an individualized anesthesia plan already set in motion. Antenatal anesthesia clinics in the U.S. and U.K. have reported similar utility gained [15, 17].

Concurrent cardiac disease occurs in approximately 1% of pregnant women [20]. Among the 72,000 deliveries managed in Hadassah since establishment of the clinic, cardiac disease would be expected in about 7000 women. Yet only 66 women with cardiac disease were referred, raising the possibility that many others were not referred and did not undergo optimized labor preparations. A similar phenomenon was reported in a neighboring medical center in Jerusalem [16], concurring with reports from other countries on non-referral of pregnant women despite the potential for benefit [12, 15–17]. We know that many women who delivered in our institution had cardiac conditions; but these were not charted. Thus the precise frequency of underlying cardiac conditions in our population is unknown.

Women view delivery as a unique experience and are likely have specific analgesia plans in mind. It is important to avoid disappointment, particularly if neuraxial analgesia is desired [21]. Our clinic provided pregnant women who were unsure if neuraxial block was possible due to a hematologic condition, with an alternative analgesia options for labor.

Last-minute anesthesia planning and management of complicated laboring women, which was avoided for women seen in our clinic, can manifest as far more than a nuisance during delivery. It can be associated with maternal deaths [20, 22]. The UK CAPS study reported that almost a quarter of maternal cardiac arrests occurred purely as a consequence of anesthesia complications [22].

With deaths associated with cardiac or neurological disease, so-called indirect causes of maternal death, overtaking traditional causes for maternal morbidity and mortality globally, efforts should be focused on women with concurrent medical conditions [1, 3, 23]. One in ten laboring women in developed countries has at least one concurrent medical condition [3, 24]. There is no reason to assume that this figure differs in Israel. If anything, the chance of complications during pregnancy in the lifetime of an Israeli woman with high fertility rates [25] may be expected to be higher, compounded by easily accessible assisted reproduction and advanced maternal age pregnancies [26].

Rather than investigate the frequencies and impact of concurrent medical conditions on maternal labor outcomes, the obstetrical community in Israel parades the low maternal mortality rates [27] as proof of admirable care. Israel does not have or use maternal morbidity reports, thus lacks a mechanism to record or report morbidities according to WHO near-miss criteria [6]. It seems clear that this reflects the lack of awareness in our region towards this major global issue of maternal health.

The current Israeli antenatal model is comprised of two distinct pathways wherein medical information is sequestered; HMO community physicians provide antenatal care and hospital physicians provide care for labor and delivery. These frequently disconnected pathways embody the fragmentation of care that contributes to adverse maternal outcomes [2]. Antenatal anesthesia clinics function as a bridge between these pathways. Fifteen years after the opening of Israel’s first antenatal anesthesia clinic in Hadassah, most currently running clinics are in the center of the country, two are in the north and none in the south of Israel.

Given that 27 labor and delivery units between them manage approximately 180, 000 annual deliveries in Israel, and the 10% prevalence of maternal concurrent conditions, about18, 000 women per year throughout the country likely should be assessed prior to labor and delivery. This requires sufficient new clinics to handle these women. The location of such clinics should take into consideration accessibility for pregnant women, the availability of specialty consults, and options for further investigations; and this may vary according to the availability of these services, geographical limitations and cultural sensitivities. Regardless of clinic location all information on maternal medical conditions must be communicated to the hospital medical staff. This should occur when the woman selects her delivery hospital rather than at the time of delivery. Furthermore, Israel is one of the first countries in the world to have a certified Obstetric Anesthesia Fellowship program. These experts should be active partners in the antenatal high-risk clinics.

As expected, musculoskeletal conditions were the foremost reason for referral our clinic, as they comprised 30% of referrals in another Israeli antenatal anesthesia clinic [16], and 60% in a Canadian clinic [28]. Despite being a prevalent cause of concern for pregnant women, musculoskeletal conditions are missing from the 2011 MOH list [18]. Other significant reasons for referrals were contraindications to neuraxial anesthesia, prior severe allergic reactions, anticipated difficult airway management and severe neurologic conditions (e.g. multiple sclerosis, myasthenia gravis) all of which potentially endanger the mother and were not included on the MOH list. The list of conditions published in the 2017 ACOG Practice Bulletin [10] includes musculoskeletal conditions. The results of our study indicate that the Israeli Ministry of Health should update its 2011 memo to reflect the prevalence of concurrent medical conditions.

Current antenatal assessments focus predominantly on potential fetal concerns rather than maternal ones [7, 18]. The HMOs know when a woman is pregnant, and using health information technology should know if she has a relevant medical condition. If this is the case, a referral can be generated to the antenatal clinic who will have a responsibility to refer her for anesthesia evaluation. The high-risk antenatal services in the HMO and hospitals should be providing care for mothers at risk. The World Health Organization (WHO) acknowledges in their 2016 recommendation of antenatal care that women are the best custodians of their medical information. The paper copy of their pregnancy details that women carry [29] should also contain thorough and pertinent medical details.

Once a pregnancy in a woman with concurrent medical conditions has been flagged by her clinician, completion of a medical checklist may be set as a prerequisite to access to care. An antenatal care checklist was introduced in 2001 by the WHO, and such a list should accompany women’s pregnancy and delivery care. The Israeli model checklist should be generated based both upon the prevalence of concurrent medical conditions, and potential maternal morbidities. It should be generated by a multidisciplinary task force of obstetricians, HMO physicians, anesthesiologists and intensive care physicians. The primary reason to create checklists is to ensure a safety net for maternal care within the correct framework. This would ensure referral to an obstetrician experienced in management of high risk women. We therefore propose that the high risk antenatal clinic flag the mother for one or both pathways: maternal concerns, led by an obstetric anesthesiologist and involving obstetrics, anesthesia, and expert consults; and obstetric/fetal risk, managed as is done today. A secondary advantage of having a mandatory computerized medical checklist is the creation of the registry needed to inform future policy decisions. In addition, selective referral to specialist care can cause anxiety [30]. Thus, another benefit of establishing standard referral pathways is the opportunity to overcome concerns and improve compliance by establishing that routine excellent care involves referral of mothers in specific instances to ensure the best pregnancy outcome.

Attending pre-anesthesia clinics has been demonstrated to be cost-beneficial for non-pregnant patients. Relevant benefits observed include reduced morbidity and increased efficiency and patient satisfaction [11, 31]. However, similar to other studies of antenatal anesthesia clinics [15], we do not have the data at this time to report cost benefit. Nonetheless, we are aware anecdotally of women who may have benefited from the antenatal anesthesia clinic.

One major study limitation is that we do not know the frequencies of concurrent morbidities of women who did not attend the antenatal anesthesia clinic in our laboring population. Concurrent medical conditions were not recorded reliably in our labor ward EMR and when listed were not according to the International Disease Classification. Thus, we do not know the number of women with concurrent morbidities who should have been referred to the antenatal anesthesia clinic. The only robust medical condition information available was our data from the antenatal anesthesia clinic. In addition, we do not include women hospitalized antenatally with concurrent medical conditions who were assessed by an anesthesiologist during the in-patient period.

Most medical centers, including Hadassah, have a preoperative anesthesia clinic to assess pregnant women who are planning elective cesarean delivery. Although we are not able to report these data, some women with concurrent medical conditions may have been assessed in this setting. Such a clinic could be extended to include all women with concurrent medical conditions who need antenatal anesthesia evaluations. However often this is an ad hoc service performed by over-stretched dedicated labor ward anesthesiologists, possibly performed by junior staff, and takes place close to the date of surgery. Most women in our cohort attended the antenatal anesthesia clinic in the early third trimester, leaving ample time for additional consults and investigations. Although flyers and personal letters were sent several times to the gynecologists and primary physicians servicing the Jerusalem area about the Hadassah antenatal anesthesia clinic, we do not know their awareness in the community of this service.

Multidisciplinary consults were performed on an ad hoc basis and we lack data regarding referrals to other medical disciplines. Some women presented with a specialist’s opinion in writing; this process should be refined to ensure specialists consider also anesthesia considerations when giving expert advice. In the past 12 months we have instituted an antenatal cardiac anesthesia clinic (cardiologists, anesthesiologists, intensivists, obstetricians, neonatologists, pulmonologists, and cardiothoracic surgeons) in order to streamline care for women with concurrent cardiac conditions. We did not assess the awareness of obstetricians regarding the importance of antenatal anesthesia assessment of women with concurrent medical conditions.

Although we report a wide range of morbidities seen in the clinic, ours is probably a biased sample. Women who attended the clinics were from all four Israeli HMOs, yet most were from the major Jerusalem HMOs, Maccabi and Meuhedet which does not reflect the prevalence of HMO membership of the country as a whole. It is not possible to know if this represents referral bias or a higher population of women with concurrent medical conditions in those two HMOs. We believe that it is likely that the prevalence of concurrent conditions is similar across HMOs, and we also believe that our study in the Jerusalem decrease the strength of the conclusions we draw for the country.

Finally, although we know that the epidural analgesia rate during the study period was 60%, unfortunately we do not have additional data on the frequency of use of alternative analgesia techniques in the laboring population of Hadassah Medical Center.

## Conclusions

Delivery cannot be postponed. But it can be managed either well or poorly. In the latter case, excellent “just-in-time” in-hospital care can deflect death, but questions remain regarding morbidity implications. Furthermore, chaos may be associated with the need to develop last minute plans for managing a pregnant woman with concurrent medical conditions who lacked antenatal anesthesia preparation. This chaos can, and should, be avoided. The current Israeli economic model of antenatal care is fragmented and does not support early assessment of maternal concurrent medical conditions despite the Ministry of Health’s 2011 memorandum. An ad-hoc referral system from HMOs to hospital antenatal anesthesia clinics is inadequate.

From a societal perspective, early assessment should be cheaper. Future research should be directed to determining the cost-effectiveness of pathways of care for these mothers and their outcomes. We have suggested one possible way to identify women with concurrent medical conditions at an early stage of pregnancy, and to streamline their care with early multidisciplinary planning. Women with concurrent medical conditions should have labor and anesthesia plans considered during the nine months of pregnancy, prior to delivery, and hospitals should have a means of obtaining this information in a timely manner. Finally, there is a need to develop additional antenatal anesthesia clinics.

## Abbreviations

ACOG: American College of Obstetricians and Gynecologists
CHD: congenital heart disease
EMR: electronic medical record
HMO: health maintenance organization
IV: intravenous
PCA: patient controlled analgesia
PPH: postpartum hemorrhage
RF: rheumatic fever
